# Metal–Organic Framework-Immobilized Mycotoxin-Degrading Enzymes: Interfaces, Host Design, and Food/Feed Applications

**DOI:** 10.3390/toxins18080353

**Published:** 2026-08-19

**Authors:** Boyu Fang, Miao Long

**Affiliations:** Key Laboratory of Livestock Infectious Diseases, Ministry of Education, College of Animal Science and Veterinary Medicine, Shenyang Agricultural University, 120 Dongling Road, Shenyang 110866, China; 2024240850@stu.syau.edu.cn

**Keywords:** mycotoxin control, enzymatic detoxification, metal–organic frameworks, enzyme immobilization, MOF–enzyme interfaces, food and feed safety

## Abstract

Mycotoxin contamination remains a persistent threat to food and feed safety owing to the chemical stability of many mycotoxins, frequent co-occurrence, and matrix-dependent risks. Enzymatic detoxification enables structure-targeted transformation of toxicity-determining motifs, such as epoxide rings, reactive double bonds, amide linkages, and lactone structures. However, free mycotoxin-degrading enzymes are often constrained by poor operational stability, difficult recovery, and limited adaptability to complex matrices. Metal–organic frameworks (MOFs) provide programmable microenvironments for enzyme immobilization through tunable pore structures, interfacial chemistry, and confinement effects. This review links toxic structural motifs with enzymatic transformation targets, discusses MOF–enzyme interface engineering and representative host–enzyme compatibility, and evaluates application modes including single-enzyme systems, multi-enzyme co-immobilization or cascade systems, adsorption–degradation coupling, and detection–degradation integration. Key bottlenecks involving enzyme leakage, mass-transfer limitation, real-matrix stability, scalable preparation, and biosafety are critically discussed. Rather than treating MOFs as passive enzyme carriers, this review proposes an application-oriented framework that integrates toxin structure, enzyme function, MOF interface regulation, matrix compatibility, and safety validation to guide the development of MOF-immobilized degrading enzymes for practical mycotoxin detoxification.

## 1. Mycotoxin Contamination, Structural Toxicity, and Detoxification Demand

Mycotoxin contamination represents a persistent global challenge to food and feed safety because many mycotoxins are chemically stable, frequently co-occur in contaminated matrices, and are difficult to eliminate once formed. These features indicate that effective detoxification should move beyond reductions in toxin concentrations and instead consider structure-targeted transformation, matrix compatibility, and safety evaluation. Against this background, animal feed represents a particularly important scenario for mycotoxin control.

**Figure 1:**
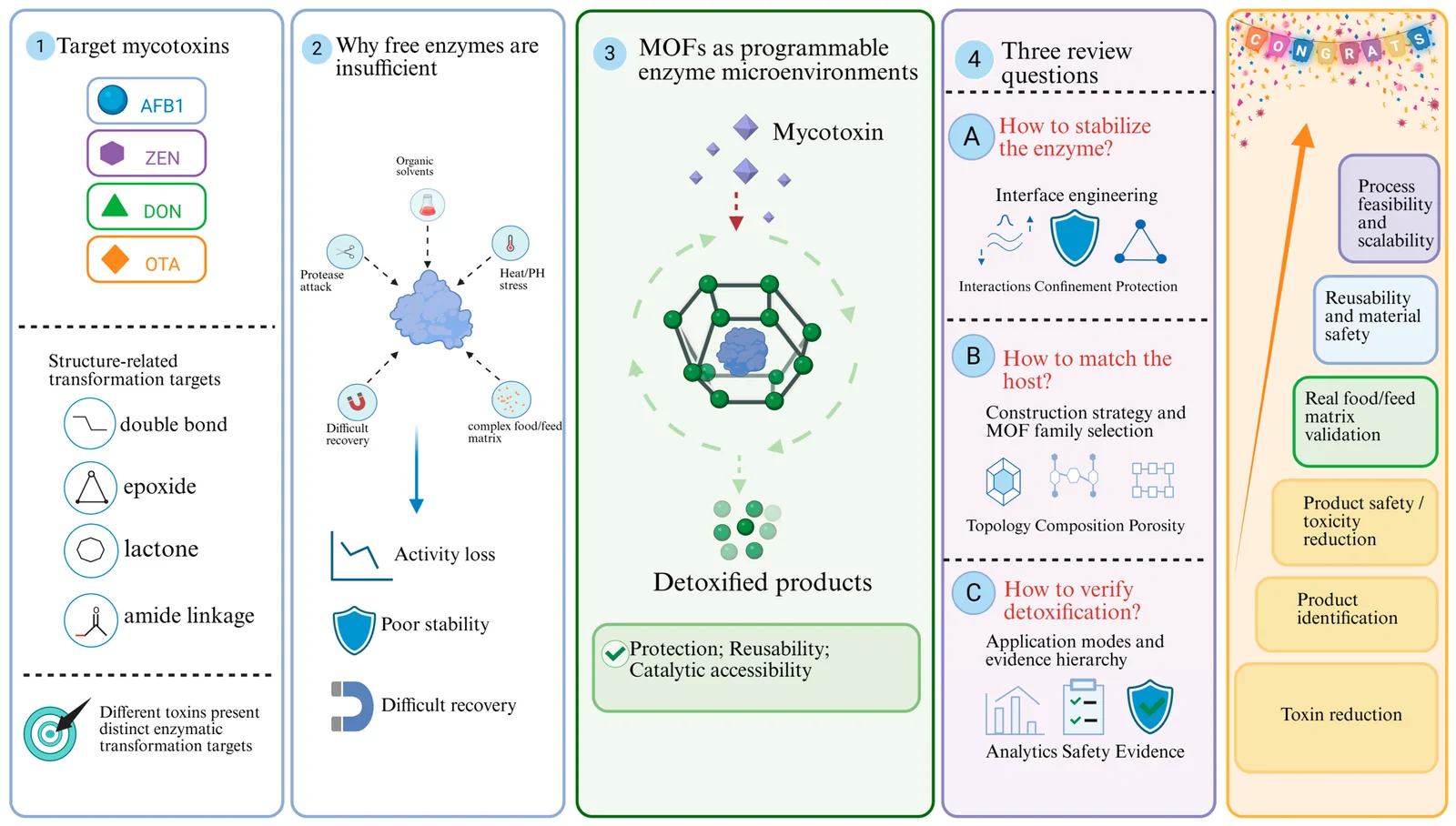
Conceptual roadmap for MOF-based immobilization of mycotoxin-degrading enzymes toward application-oriented detoxification.

Animal feed is highly susceptible to fungal contamination during storage and transportation, particularly under humid conditions. Mycotoxins are secondary fungal metabolites that can seriously impair feed quality and animal health. Common mycotoxins include deoxynivalenol (DON), aflatoxin B1 (AFB1), zearalenone (ZEN), and ochratoxin A (OTA) [1]. Among them, DON is considered one of the more hazardous mycotoxins because of its stable chemical structure. Ingestion of DON can cause diarrhea, vomiting, and gastrointestinal injury, and in severe cases may cause severe systemic effects under high-level exposure [2]. As a representative aflatoxin, AFB1 also causes substantial economic losses in livestock and poultry production and exhibits pronounced hepatotoxicity and nephrotoxicity; it may also lead to abnormal fetal development or fetal death [3]. ZEN, a Fusarium-derived feed contaminant, has estrogen-like structural features. As a result, in addition to causing hepatic and intestinal toxicity, it may also damage estrogen-target organs such as the ovaries [4]. OTA primarily exhibits nephrotoxicity, including renal fibrosis, atrophy, and cellular injury [5]. The complex composition of feed matrices and the chemical stability of mycotoxins limit the efficiency of conventional detoxification methods, which may also affect feed palatability. Accordingly, the development of novel detoxification strategies requires a deeper understanding of the structural characteristics of mycotoxins, so that targeted transformation approaches can be designed. The conceptual progression from toxicity-associated structural motifs and free-enzyme limitations to MOF-enabled immobilization and application-oriented validation is presented in Figure 1.

Representative mycotoxins, including AFB1, ZEN, DON, and OTA, contain distinct structure-related transformation targets, such as double bonds, epoxide groups, lactone motifs, and amide linkages. Although free degrading enzymes can mediate structure-directed detoxification, their practical performance in food and feed systems is often limited by heat/pH stress, protease attack, organic solvents, and complex matrix interference, leading to activity loss, poor stability, difficult recovery, and weak matrix adaptability. MOFs can function as programmable enzyme microenvironments. Through interfacial regulation, confinement protection, and mass-transfer control, they may improve enzyme protection, catalytic accessibility, reusability, and matrix adaptability. This review is organized around three key questions: how to stabilize the enzymes, how to match the MOF hosts, and how to verify detoxification. Application-oriented evaluation should begin with parent-toxin reduction and then verify product identity, toxicity attenuation, real-matrix performance, catalyst reuse, material safety, and process feasibility.

**Figure 2:**
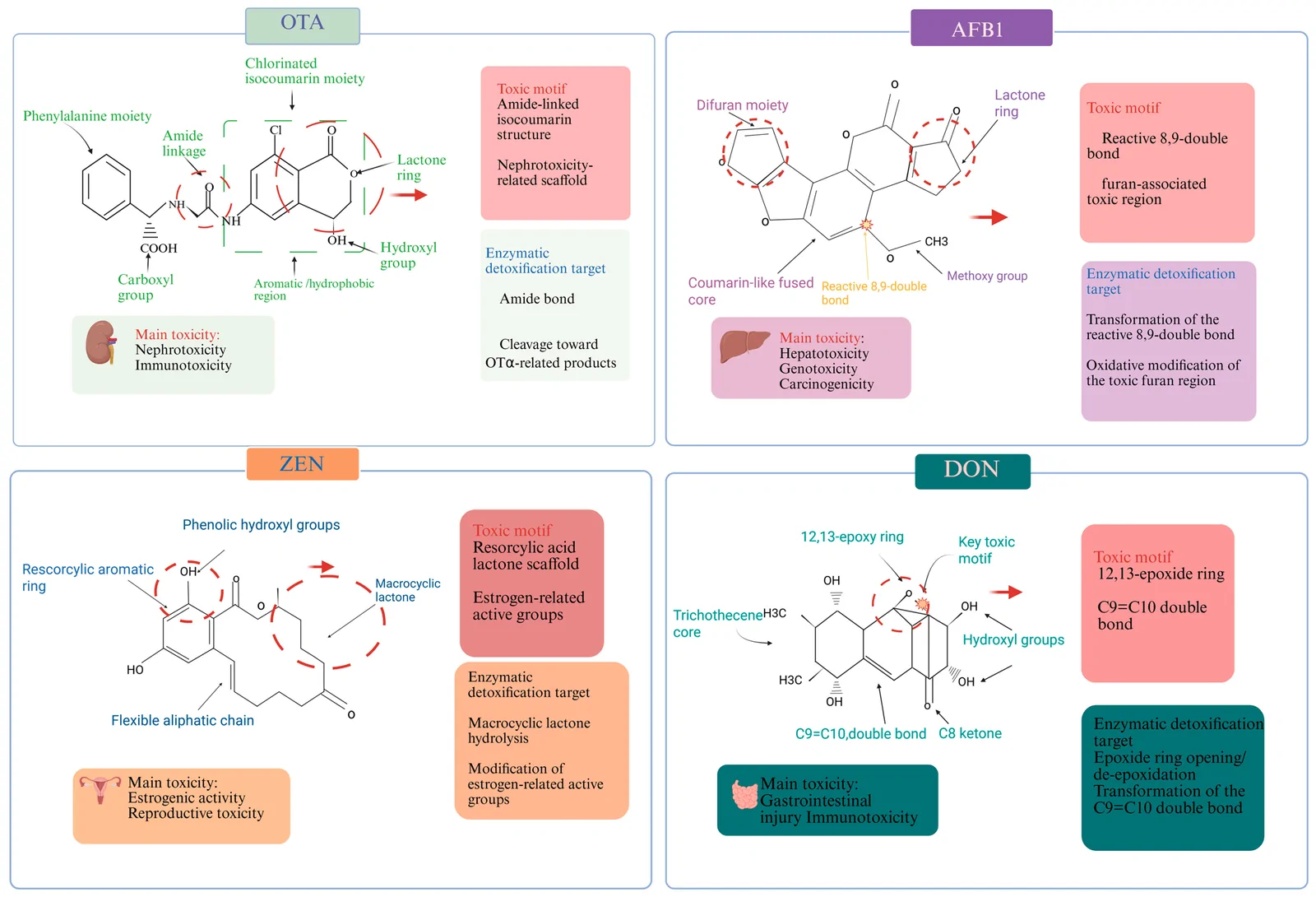
Key structural features associated with the toxicity and enzymatic transformation of representative mycotoxins.

At the molecular level, DON toxicity is closely associated with the 12,13-epoxide ring and the C9=C10 double bond. The oxygen atom in the 12,13-epoxide ring can form a key hydrogen bond with the 2′-hydroxyl group of U2873 ribose in yeast 25S rRNA, while the C9=C10 double bond and its ring system can interact with the cytosine ring of C2821 and the adenine ring of A2820 through hydrophobic stacking [6]. Therefore, structural disruption of the epoxide ring or modification of the C9=C10 double bond can markedly influence DON toxicity [7]. Reactive molecular dynamics simulations further showed that the reaction between DON and reactive oxygen species may induce epoxide ring opening and double-bond addition/cleavage, suggesting attenuation of toxicity-related structural features [8]. For AFB1, the terminal furan C8=C9 double bond is a key structural basis for metabolic activation. CYP450-mediated epoxidation produces AFB1-exo-8,9-epoxide (AFBO), which can react with DNA and contribute to mutagenic and carcinogenic effects, while CYP450-related oxidative stress may further aggravate DNA damage through ROS generation [9]. Studies using CYP1A2/CYP3A4 inhibitors or multivalent aptamers further support the importance of this structural site: Tsega et al. reported that catechin and quercitrin could mitigate AFB1-induced cytotoxicity by inhibiting CYP1A2 and CYP3A4, whereas Chang et al. showed that a polyvalent aptamer could cover key toxic sites of AFB1, including the furan-ring C8=C9 double bond, thereby reducing AFBO formation and alleviating AFB1-induced toxicity [10,11]. ZEN toxicity is mainly attributed to its estrogen-like resorcylic acid lactone scaffold, in which phenolic hydroxyl groups and lactone-related structures contribute to estrogen receptor binding and endocrine-disrupting activity [12]. These examples indicate that mycotoxin detoxification should be understood as the structure-directed transformation of toxicity-associated motifs. Enzymatic approaches, including oxidoreductases, hydrolases, and transferases, can target reactive epoxide rings, double bonds, amide linkages, lactone structures, and other toxic functional groups, thereby providing a molecular basis for enzyme-mediated detoxification and subsequent MOF-based immobilization design [13]. Figure 2 further illustrates how the characteristic structural motifs of representative mycotoxins define the potential targets for enzymatic transformation.

Ochratoxin A (OTA) contains an isocoumarin moiety linked to L-phenylalanine through an amide bond and exhibits prominent nephrotoxicity [5]. Enzymatic hydrolysis of this amide bond, yielding ochratoxin α and phenylalanine, represents a documented OTA transformation pathway [14,15]. The toxicity of aflatoxin B1 (AFB1) is strongly associated with the terminal furan C8=C9 double bond, which undergoes cytochrome P450-mediated epoxidation to form the highly reactive AFB1-exo-8,9-epoxide [9]. Deoxynivalenol (DON) contains a trichothecene core in which the 12,13-epoxide ring and C9=C10 double bond are important determinants of ribosome binding and toxicity [6,7]. Zearalenone (ZEN) is a resorcylic acid lactone mycotoxin whose estrogenic activity is associated with its interaction with estrogen receptors [4,12]. These structure–toxicity relationships provide the molecular basis for enzymatic detoxification through reactions such as oxidation–reduction, epimerization, amide-bond hydrolysis, and lactone-ring cleavage [13,16,17].

Accordingly, enzymatic detoxification can be viewed as the targeted transformation of toxicity-determining structural motifs, which may include epoxide ring opening, double-bond modification, amide-bond hydrolysis, and lactone-ring cleavage. This structure-oriented perspective provides the molecular basis for enzyme-mediated detoxification and further indicates that immobilization platforms may be designed according to both the structural features of target toxins and the catalytic modes of the corresponding enzymes. Table 1 brings these relationships together by linking the key toxic structural motifs of representative mycotoxins with their enzymatic transformation routes, corresponding MOF design considerations, and safety-evaluation priorities.

Although MOF-based enzyme immobilization has been widely discussed in general biocatalysis, its translation to mycotoxin detoxification requires a more specific framework. Mycotoxin detoxification depends not only on enzyme loading or catalytic activity, but also on toxin-specific structural motifs, transformation-product safety, matrix interference, and material residues. Thus, this review focuses on MOF-immobilized mycotoxin-degrading enzymes from an application-oriented perspective, emphasizing the interplay among toxic structural motifs, enzymatic transformation targets, MOF-regulated interfaces, real food and feed matrices, and safety validation. This perspective distinguishes the present review from general enzyme@MOF reviews and establishes a basis for assessing MOFs as programmable enzyme microenvironments for practical food and feed detoxification.

## 2. Enzymatic Detoxification of Mycotoxins: Catalytic Potential and Application Bottlenecks

Conventional physical detoxification methods, such as separation, washing, milling, heating, and irradiation, can reduce mycotoxin levels to some extent, while chemical detoxification generally relies on oxidation, hydrolysis, or alkaline treatment to disrupt toxin structures [27,28]. Their application may nevertheless be limited by incomplete detoxification, nutrient loss, altered palatability, and uncertainty regarding transformation products. Enzymatic detoxification offers a more selective alternative because degrading enzymes can transform toxicity-associated structural motifs under relatively mild conditions [29,30]. Microorganisms remain an important source of mycotoxin-degrading enzymes. Screening strategies often use mycotoxins or their structural analogues to enrich strains capable of toxin transformation, and the resulting degradation activity may be mediated by extracellular enzymes, intracellular enzymes, or other bioactive components [29,30]. From a catalytic perspective, the major enzyme types involved in mycotoxin detoxification include oxidoreductases, hydrolases, and transferases, which can mediate reactions such as oxidation, reduction, hydrolysis, ring opening, and functional-group modification [13,30]. Accordingly, the value of enzymatic detoxification lies not only in lowering toxin concentration, but also in selectively transforming toxicity-determining structural motifs.

The practical use of free mycotoxin-degrading enzymes remains limited by their sensitivity to temperature, pH, organic solvents, proteases, and matrix components [31,32,33,34]. In food and feed systems, these factors can alter enzyme conformation and reduce substrate accessibility, making buffer-based activity an unreliable predictor of application performance. Protein engineering can improve intrinsic catalytic properties; for example, structure-guided modification has enhanced OTA-degrading amidohydrolases and the acidic performance of zearalenone lactonases [35,36]. However, such approaches do not directly address enzyme recovery, reuse, or retention within the treated system.

Immobilization is therefore required when operational stability and catalyst recovery are as important as intrinsic activity. Conventional carriers, including chitosan- and liposome-based systems, can provide partial improvements in stability or controlled release [37,38,39]. MOFs offer an additional level of control through tunable pore architecture, surface chemistry, and confinement [40]. The following section examines how these features can be matched with enzyme, toxin, and matrix requirements.

## 3. MOFs as Programmable Enzyme Microenvironments for Mycotoxin Detoxification

Free mycotoxin-degrading enzymes often exhibit limited operational robustness and practical applicability under food- and feed-relevant conditions [31,32,33,34]. MOF-based immobilization should therefore be viewed not as simple enzyme attachment, but as the construction of a local catalytic microenvironment that stabilizes the enzyme without compromising catalytic access. The function of the MOF is determined by how its structural and chemical properties regulate enzyme–toxin interactions rather than by enzyme loading alone.

MOFs are crystalline porous materials assembled from metal nodes and organic linkers through reticular coordination chemistry [41,42]. Their pore architecture, linker functionality, metal-node chemistry, defect sites, and surface properties can be adjusted at the framework level [20,21,41,43]. These features influence enzyme accommodation, conformational protection, catalytic accessibility, mass transfer, and operational stability [20,21,43]. For mycotoxin detoxification, host selection should therefore be treated as a host–enzyme–toxin matching problem. The selected framework must be compatible with enzyme size and surface properties, preserve access to the catalytic site, and support the transport requirements of the target toxin and its transformation products.

A central design challenge is the balance between enzyme protection and molecular transport. Spatial confinement can reduce enzyme inactivation under thermal, solvent, proteolytic, or other denaturing conditions, but excessive confinement may restrict toxin entry, product release, or, where applicable, cofactor and mediator transport. Hierarchical mesoporous MOFs offer one possible solution by combining larger spaces for enzyme accommodation with interconnected channels for smaller molecules [44,45]. Because AFB1, ZEN, DON, and OTA differ in polarity, hydrophobicity, and molecular structure, a host suitable for one toxin–enzyme pair cannot be assumed to perform equally well for another.

Framework stability must be evaluated alongside enzyme stability. Water resistance, acid–base tolerance, thermal stability, and mechanical integrity vary substantially among MOF families [46,47]. Accordingly, framework integrity and pore accessibility should be verified under the intended application conditions rather than inferred from buffer-based performance. Immobilization mode further shapes the relationship between protection and catalytic access: surface attachment leaves the enzyme comparatively exposed, whereas biomimetic encapsulation creates a protective porous shell whose transport properties depend on framework and pore accessibility [20,48]. These host-level considerations connect framework selection with the interfacial and construction choices that ultimately determine detoxification performance.

Process-compatible preparation is another emerging consideration. Solvent-free spray drying has been used to prepare phytase@MIL-88A with enhanced thermal stability and controlled release [49]. This study supports the feasibility of process-compatible enzyme@MOF formulation but does not establish the industrial readiness of mycotoxin-degrading enzyme@MOF systems. Overall, a suitable MOF host must provide the required enzyme protection and molecular access while retaining structural integrity in the intended toxin–enzyme–matrix system.

## 4. MOF–Enzyme Interfacial Mechanisms: From Molecular Interactions to Detoxification Function

**Figure 3:**
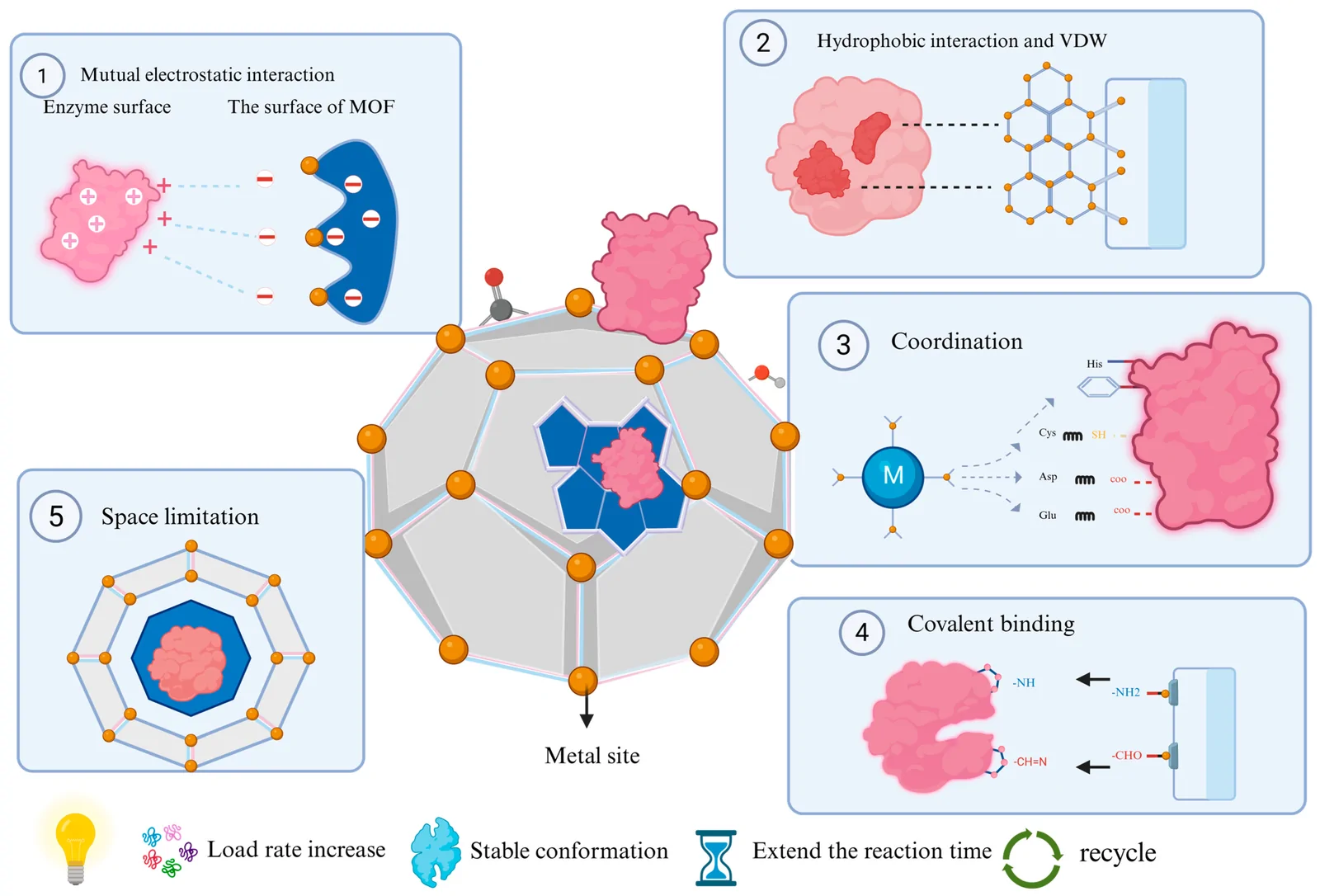
Interfacial mechanisms involved in MOF-based enzyme immobilization.

The performance of MOF-immobilized mycotoxin-degrading enzymes depends on how interfacial and spatial interactions affect enzyme anchoring, conformation, active-site exposure, and molecular transport. These interactions should therefore be regarded as functional design variables rather than merely as binding forces. The principal mechanisms and their roles in enzyme stabilization and catalytic accessibility are depicted in Figure 3.

MOF-immobilized enzymes are stabilized through multiple interfacial and spatial mechanisms, including electrostatic interactions, hydrophobic interactions and van der Waals forces, coordination interactions, covalent bonding, and spatial confinement. Electrostatic interactions mainly support charge matching and initial adsorption, whereas hydrophobic and van der Waals forces help regulate interfacial fitting and enzyme orientation. Coordination and covalent bonding can strengthen enzyme anchoring, while spatial confinement protects enzymes within MOF pores or cages. Together, these mechanisms regulate enzyme loading, conformational stability, catalytic durability, and reusability.

### 4.1. Charge-Regulated Adsorption and Buffer-Sensitive Enzyme Loading

Electrostatic interactions contribute to the initial attachment of enzymes to MOF surfaces. Because both enzymes and MOFs exhibit pH-dependent surface charges, loading is influenced by the enzyme isoelectric point, MOF surface chemistry, ionic strength, and buffer composition. Favorable charge matching can promote mild adsorption, whereas unsuitable conditions may weaken interfacial attraction and increase enzyme desorption [50,51,52].

This sensitivity is particularly relevant in food and feed applications, where salts, organic acids, proteins, polysaccharides, and lipids may alter local charge conditions or compete with enzyme–MOF interactions. Buffer composition has been shown to affect enzyme loading, ζ-potential, and catalytic performance, confirming that the reaction medium actively regulates the interface [53,54,55].

Electrostatic adsorption is therefore useful for mild immobilization but may provide insufficient long-term retention when used alone. Its effectiveness should be assessed through enzyme leakage, activity retention, and performance under relevant matrix conditions and, where necessary, reinforced by coordination, covalent anchoring, or confinement.

### 4.2. Pore-Wall Chemistry, Hydrophobic Contacts, and Enzyme Orientation

Hydrophobic interactions and van der Waals forces contribute to enzyme–surface fitting, orientation, and local conformational adaptation. Their importance lies not simply in increasing binding strength, but in determining how the enzyme contacts the pore wall and whether the catalytic site remains accessible [50,56,57,58].

Hydrophobic or amphiphilic environments may promote the local accessibility of mycotoxins such as AFB1 and ZEN. Excessive hydrophobic contact, however, may disturb the enzyme hydration shell, restrict conformational flexibility, or reduce catalytic efficiency, particularly when pore connectivity and substrate diffusion are poorly matched [59,60,61]. Pore-wall hydrophobicity must therefore be balanced with enzyme hydration and molecular transport.

Molecular simulations of lipase–ZIF-8 and enzyme binding on chemically distinct MOF surfaces further show that hydrophobicity, hydrogen bonding, van der Waals interactions, and framework structure act together to determine interfacial behavior [62,63]. Thus, pore-wall chemistry should be optimized to stabilize enzyme orientation without compromising toxin entry or product release.

### 4.3. Metal-Site Coordination and Oriented Immobilization

Coordination interactions provide a more selective immobilization route through binding between accessible MOF metal sites and coordination-capable protein groups, particularly histidine residues or engineered His-tags. Coordinatively unsaturated sites can bind imidazole groups and potentially orient enzymes more precisely than nonspecific adsorption [64,65,66].

This approach is relevant to recombinant mycotoxin-degrading enzymes because affinity tags used for purification may also serve as anchoring modules. When positioned away from the catalytic pocket, such tags may improve enzyme orientation while preserving active-site accessibility.

However, oriented immobilization does not automatically improve catalysis. Its effect depends on metal-site accessibility, tag position and flexibility, and the distance between the anchoring region and catalytic center. Buffers, phosphates, organic acids, salts, and other competing ligands may also occupy metal sites or weaken enzyme binding [55,67].

Coordination-based systems should therefore be evaluated through orientation, leakage, activity retention, framework stability, and metal-node release. Rational designs may place affinity tags away from active sites, regulate open metal sites, or combine coordination with additional stabilization when stronger retention is required.

### 4.4. Covalent Anchoring: Interfacial Robustness Versus Conformational Risk

Covalent anchoring forms stable enzyme–MOF linkages through reactive surface groups or post-synthetic modification, thereby reducing enzyme leakage and improving durability during washing, recycling, or extended catalysis [68,69,70]. It is particularly useful when repeated operation requires stronger retention than physical adsorption can provide.

Stronger attachment, however, does not necessarily improve detoxification. Uncontrolled covalent coupling may restrict enzyme flexibility, alter local conformation, or shield the catalytic pocket. Performance therefore depends on coupling chemistry, spacer length, reaction conditions, residue distribution, and the position of the immobilization site relative to the active site [70,71,72,73].

For small-molecule mycotoxins, unfavorable orientation or excessive rigidity may reduce substrate access, product release, and catalytic turnover. Covalent systems should consequently be evaluated through activity retention and kinetic behavior as well as loading and cycling stability.

Future designs should favor site-aware coupling, defined reactive groups, and flexible spacers that reduce leakage without compromising enzyme dynamics or mass transfer.

### 4.5. Spatial Confinement and Physical Encapsulation: Protection–Diffusion Trade-Off

Spatial confinement and physical encapsulation create protective microenvironments without requiring direct covalent attachment. Biomimetic mineralization and in situ encapsulation can form MOF shells or internal spaces around enzymes, improving resistance to heat, organic solvents, proteases, and other denaturing conditions [48,74].

Protection may nevertheless be accompanied by diffusion resistance. Narrow windows, thick shells, or limited internal connectivity can restrict toxin entry, product release, and cofactor or mediator transport. Hierarchical mesoporous MOFs partly address this trade-off by combining large enzyme-accommodating pores with interconnected transport channels [44,45].

This balance becomes more demanding in food and feed matrices, where matrix components may block pores or alter framework stability. Performance in buffer therefore cannot be assumed to translate directly to oils, grains, or feeds; evaluation should distinguish conformational protection from restrictions in molecular transport.

Protein localization and clustering within MOFs also influence pore accessibility and local mass transfer [75,76]. Effective confinement design should therefore control shell thickness, pore connectivity, and enzyme distribution rather than treating successful encapsulation alone as evidence of an optimized catalytic system.

### 4.6. Design Implications for Mycotoxin-Degrading Enzyme@MOF Systems

The mechanisms described above represent complementary design options rather than isolated immobilization categories. Electrostatic adsorption enables mild loading, hydrophobic contacts influence orientation and hydration, coordination can provide selective anchoring, covalent bonding improves retention, and confinement offers protection from external stress.

Their suitability depends on the toxin–enzyme–matrix combination. Toxin polarity and molecular structure must be considered together with enzyme size, surface chemistry, active-site location, catalytic mechanism, and intended application environment. A single interaction is unlikely to optimize all of these factors, and excessive interface strength or confinement may be as detrimental as insufficient retention.

Interface design should therefore shift from maximizing loading or binding strength toward balancing enzyme retention, catalytic accessibility, molecular transport, and matrix stability. This function-oriented perspective provides the basis for selecting construction strategies and MOF hosts in Section 5.

## 5. Construction Strategies, Host Selection, and Application Modes of MOF-Immobilized Mycotoxin-Degrading Enzymes

**Figure 4:**
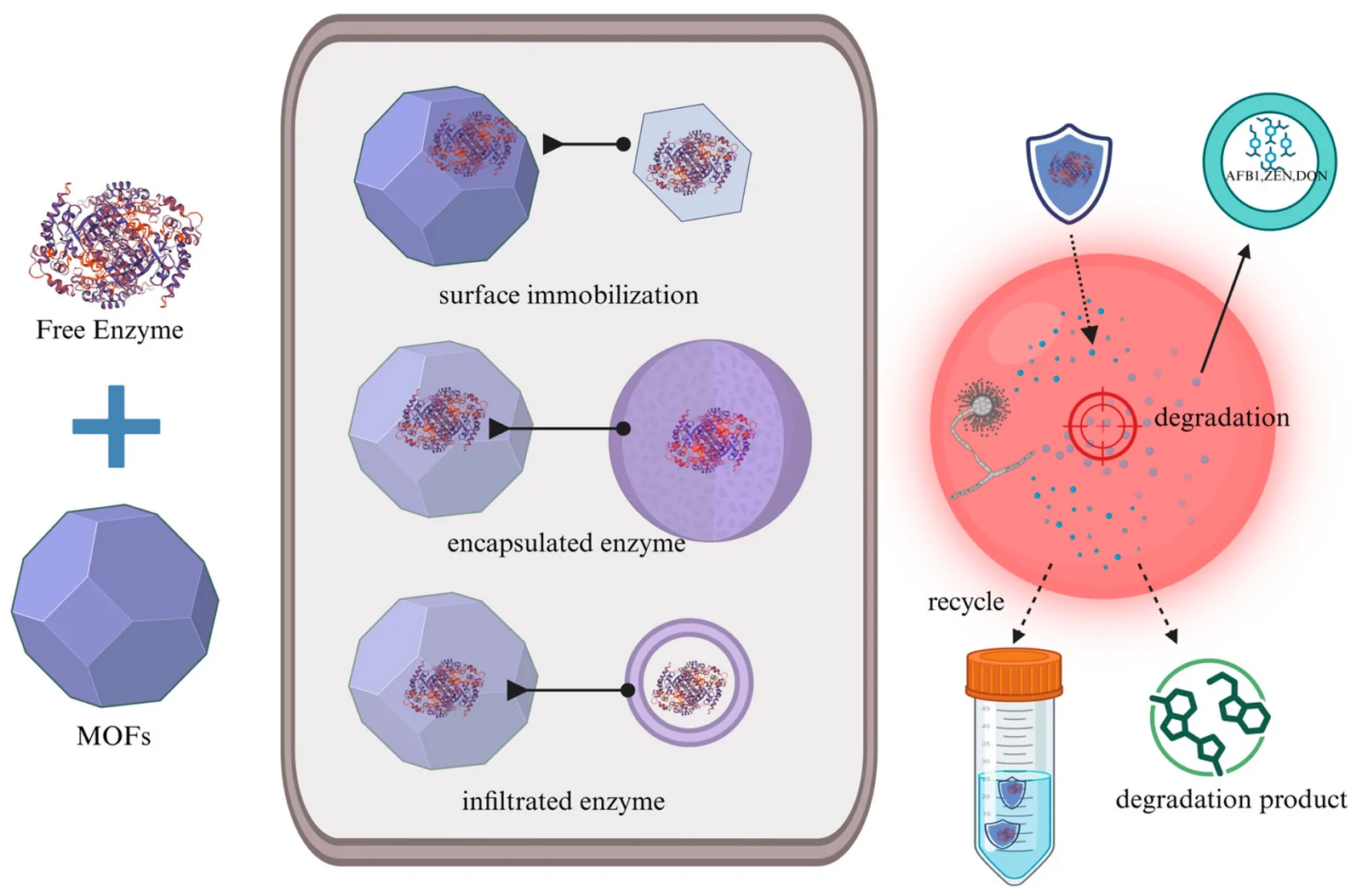
Representative immobilization modes and application-oriented functions of MOF-supported mycotoxin-degrading enzymes. Enzymes can be integrated with MOF hosts through in situ encapsulation, pore infiltration, or surface immobilization. These configurations influence enzyme protection, catalytic accessibility, molecular transport, retention, and catalyst recovery, thereby shaping mycotoxin transformation and operational reuse. The classification of the representative immobilization modes was adapted from Li et al. [77].

Figure 4 schematically summarizes the design-to-application framework of MOF-immobilized mycotoxin-degrading enzymes, linking immobilization architecture and host-framework properties with catalytic performance and practical use. The behavior of these systems arises from the coupled effects of enzyme–framework interactions, molecular transport, matrix compatibility, and operational stability, which determine whether the advantages observed under model conditions can be maintained in food and feed environments. Building on this framework, this section examines the development and application-level evaluation of enzyme@MOF systems, with emphasis on the evidence required for reliable and safe mycotoxin control.

### 5.1. Construction Strategies: Matching Immobilization Mode with Enzyme and Matrix Requirements

The construction strategy determines the spatial relationship between the enzyme and the MOF host and thereby influences catalytic exposure, molecular transport, enzyme retention, and catalyst recovery [20,21,40]. For mycotoxin-degrading systems, immobilization modes should not be selected solely according to loading capacity or ease of preparation. The more relevant question is which strategy best addresses the dominant limitation of a particular toxin–enzyme–matrix combination.

Surface immobilization is generally preferable when rapid toxin access and product release are primary requirements. Because enzymes remain exposed on the external surface of preformed MOFs, small-molecule substrates encounter fewer structural barriers than in deeply confined systems. This approach may therefore be suitable for relatively large enzymes or enzymes whose active sites must remain accessible. Its principal weakness is the possibility of enzyme desorption during washing, repeated use, or exposure to competing components in the reaction medium [20,21,40]. Dynamic exchange in preformed Zr-based MOFs has been explored as a means of strengthening enzyme retention and repeated operation in non-mycotoxin biocatalysis, although its transferability to mycotoxin-degrading systems remains to be established [78].

Coordination and covalent anchoring can reinforce surface-associated systems when enzyme leakage is a major concern, but the two strategies serve different purposes. Coordination anchoring is particularly relevant to recombinant enzymes carrying His-tags or other metal-binding motifs and may permit selective positioning on accessible MOF metal sites [65,66]. Covalent anchoring provides stronger enzyme–carrier attachment and may be preferred for repeated or prolonged operation [72,73]. In both cases, the anchoring position and coupling conditions must be controlled because stronger attachment may restrict enzyme flexibility or reduce active-site exposure. These strategies should therefore be evaluated through activity retention, leakage control, and catalytic accessibility rather than immobilization yield alone.

In situ encapsulation and biomimetic mineralization are more appropriate when enzyme deactivation is the dominant bottleneck. The formation of MOF shells or enzyme-containing frameworks can protect biomacromolecules under denaturing conditions, while recent Zr-MOF strategies have extended mild biomimetic mineralization to relatively large enzymes [48,74,75]. However, the benefit of encapsulation depends on host architecture. Narrow pore apertures, excessive shell thickness, or limited internal connectivity may restrict toxin entry, product release, and, where required, cofactor or mediator transport. Encapsulation is therefore most suitable when structural protection is essential and the selected framework can still provide adequate molecular access. Because the cited studies mainly involve model or non-mycotoxin enzymes, these advantages must be verified for individual mycotoxin-degrading enzymes and intended application matrices.

Pore infiltration and hierarchical confinement provide an intermediate strategy between external exposure and complete encapsulation. Hierarchically connected pores can accommodate enzyme molecules while maintaining pathways for smaller substrates, products, and cofactors [44,45]. This approach may be particularly useful when enzyme protection and molecular transport must be maintained simultaneously. Its success depends not only on nominal pore size, but also on pore-window dimensions, internal connectivity, enzyme distribution, and the accessibility of confined catalytic sites.

Co-immobilization and cascade construction extend this design logic to sequential transformations and cofactor-dependent reactions. MOF confinement has enabled the organization of two- or three-enzyme cascades and enzyme–cofactor components in model biocatalytic systems [79]. In mycotoxin detoxification, the co-immobilization of DADH and AKR13B3 on magnetic MOF-derived nanoflowers provides a direct example of integrating sequential DON transformation with catalyst recovery [80]. Nevertheless, the additional complexity is justified only when the assembled system provides a measurable advantage over the corresponding single-enzyme or freely mixed controls. Enzyme ratios, catalytic compatibility, intermediate accumulation, cofactor or mediator transport, and final-product safety should therefore be evaluated, particularly in real food and feed matrices.

Construction strategy selection should ultimately follow a bottleneck-oriented rather than material-centered logic. Surface immobilization is favored when molecular accessibility dominates; coordination or covalent anchoring is useful when enzyme leakage limits reuse; encapsulation is appropriate when structural protection is the primary requirement; and hierarchical or multi-component systems are warranted when protection, transport, or sequential catalysis must be coordinated. Table 2 summarizes the best-fit enzyme systems, principal advantages, key limitations, and potential detoxification scenarios associated with these construction approaches.

### 5.2. Host Selection Logic: Matching MOF Families with Detoxification Requirements

**Figure 5:**
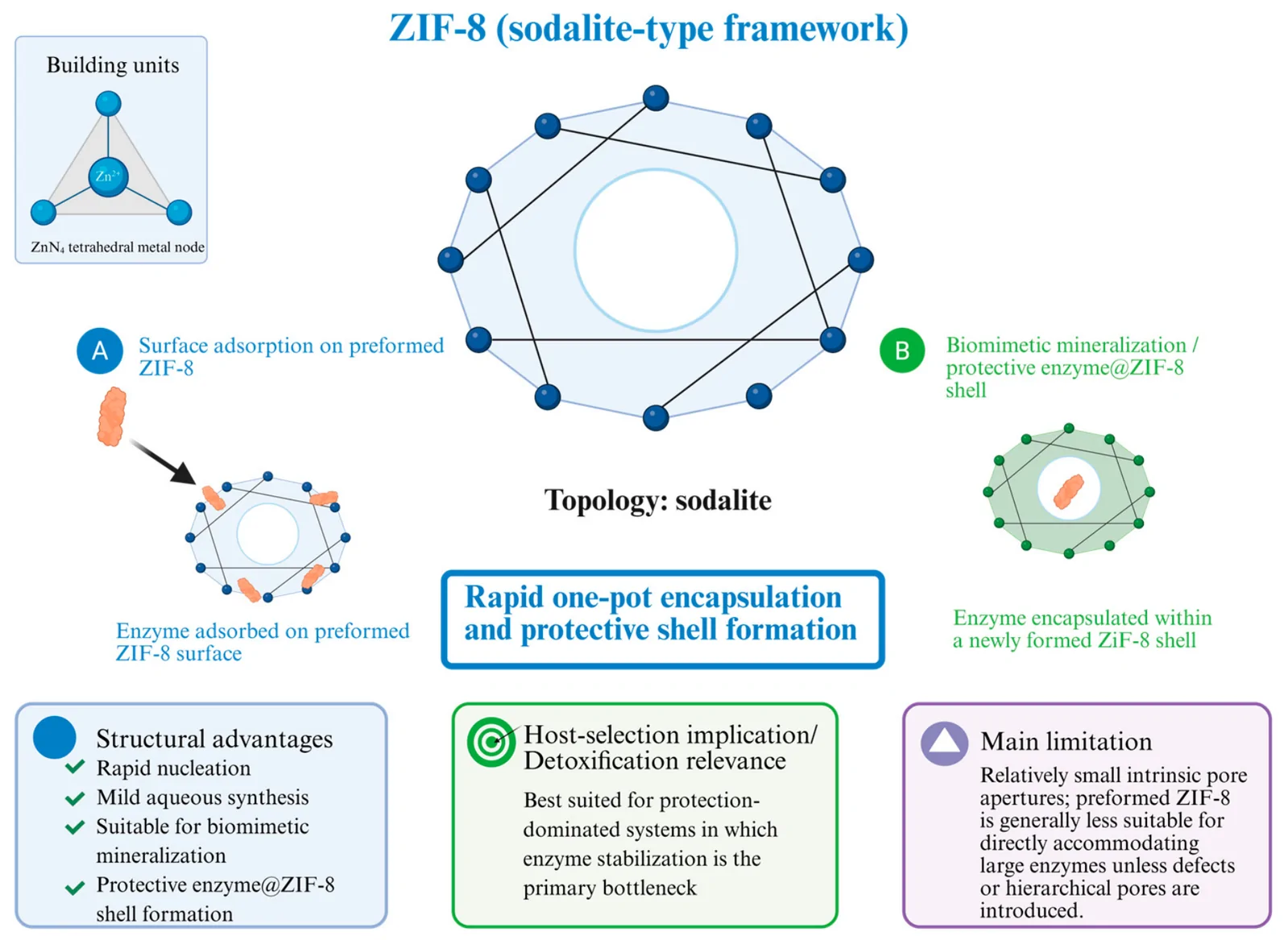
ZIF-8-type hosts for rapid enzyme encapsulation and biomimetic mineralization.

**Figure 6:**
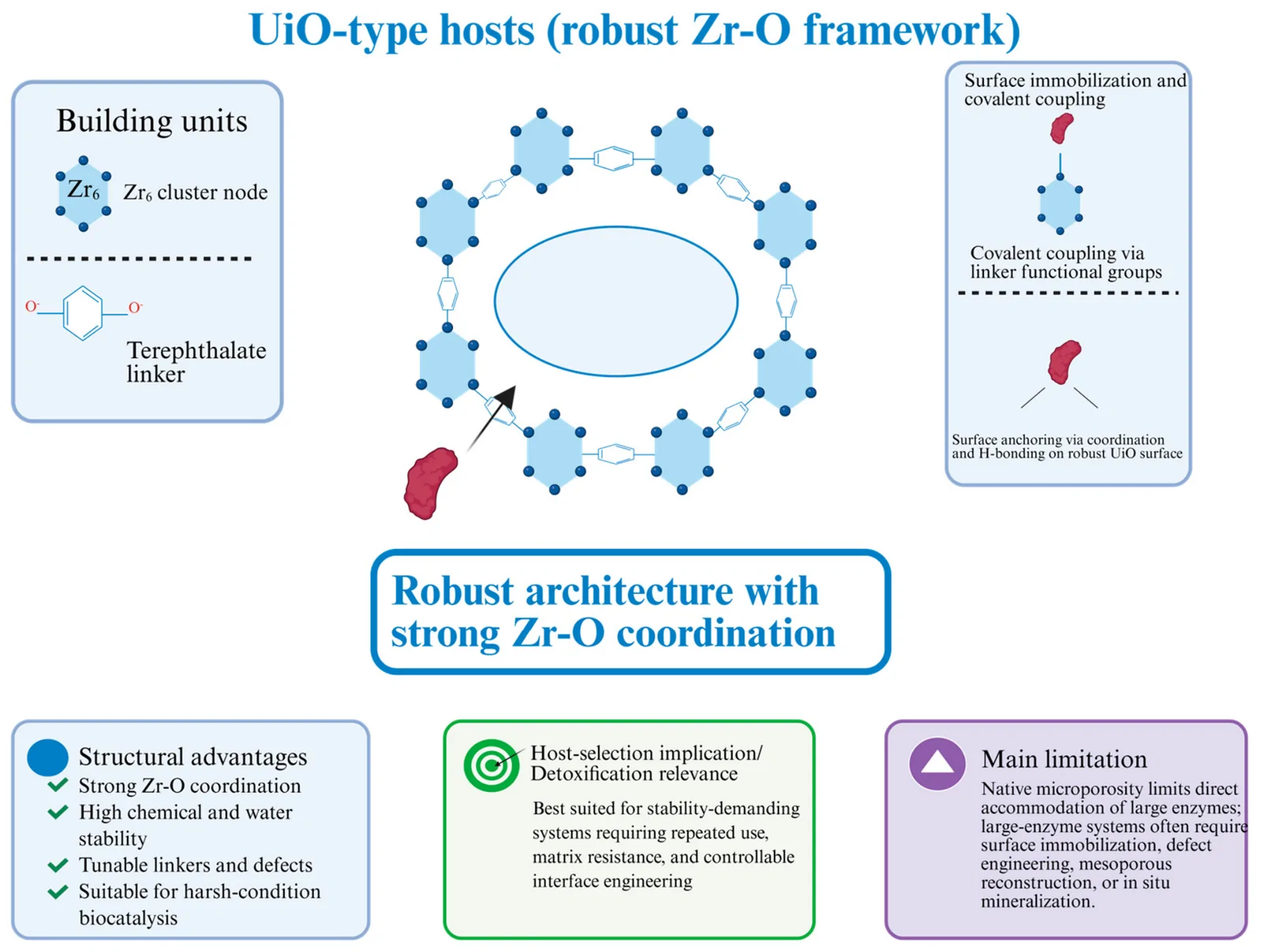
UiO-type hosts for stability-oriented enzyme immobilization and interface engineering.

**Figure 7:**
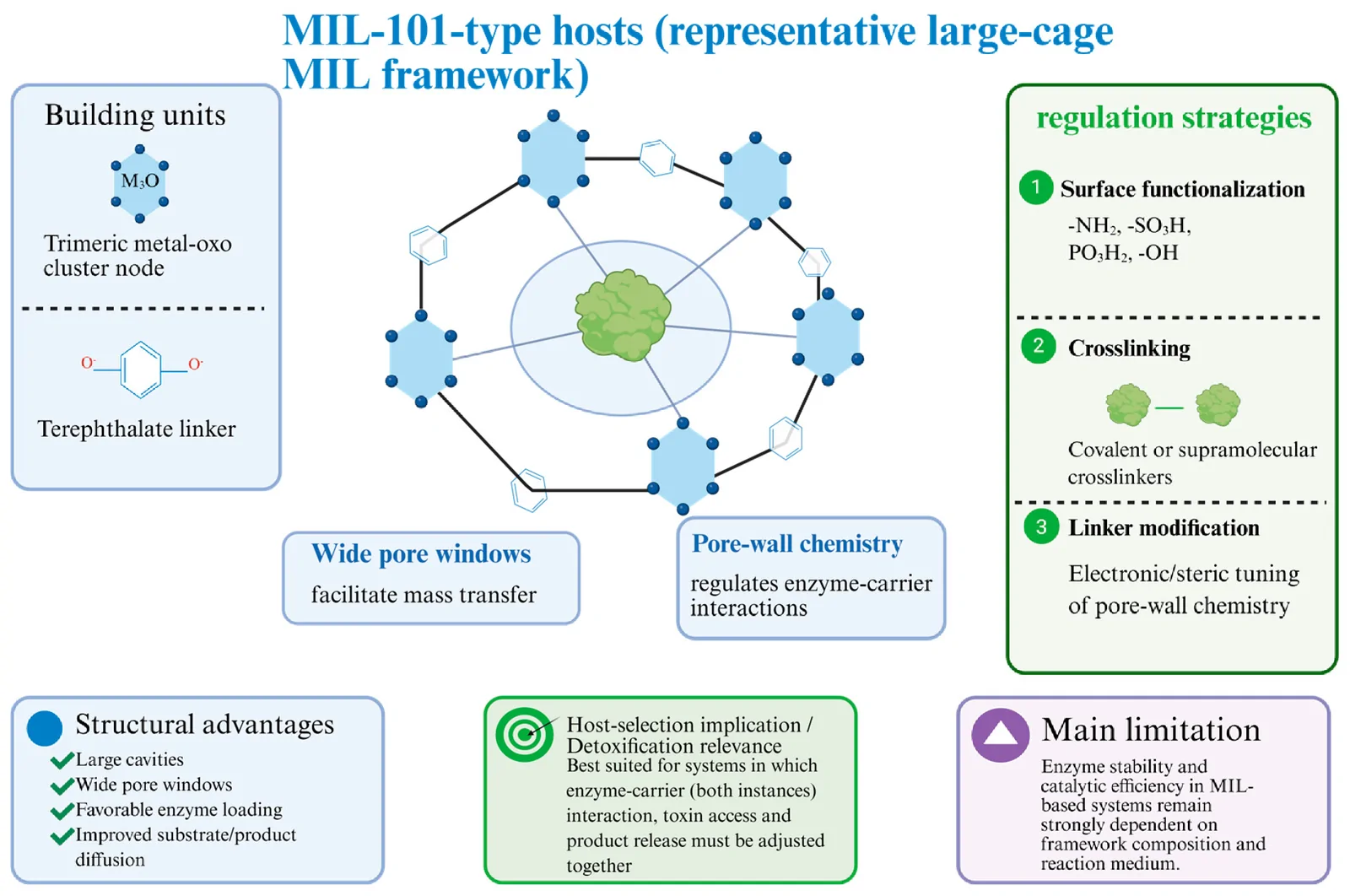
MIL-type hosts for interfacial regulation, enzyme loading, and substrate/product diffusion.

**Figure 8:**
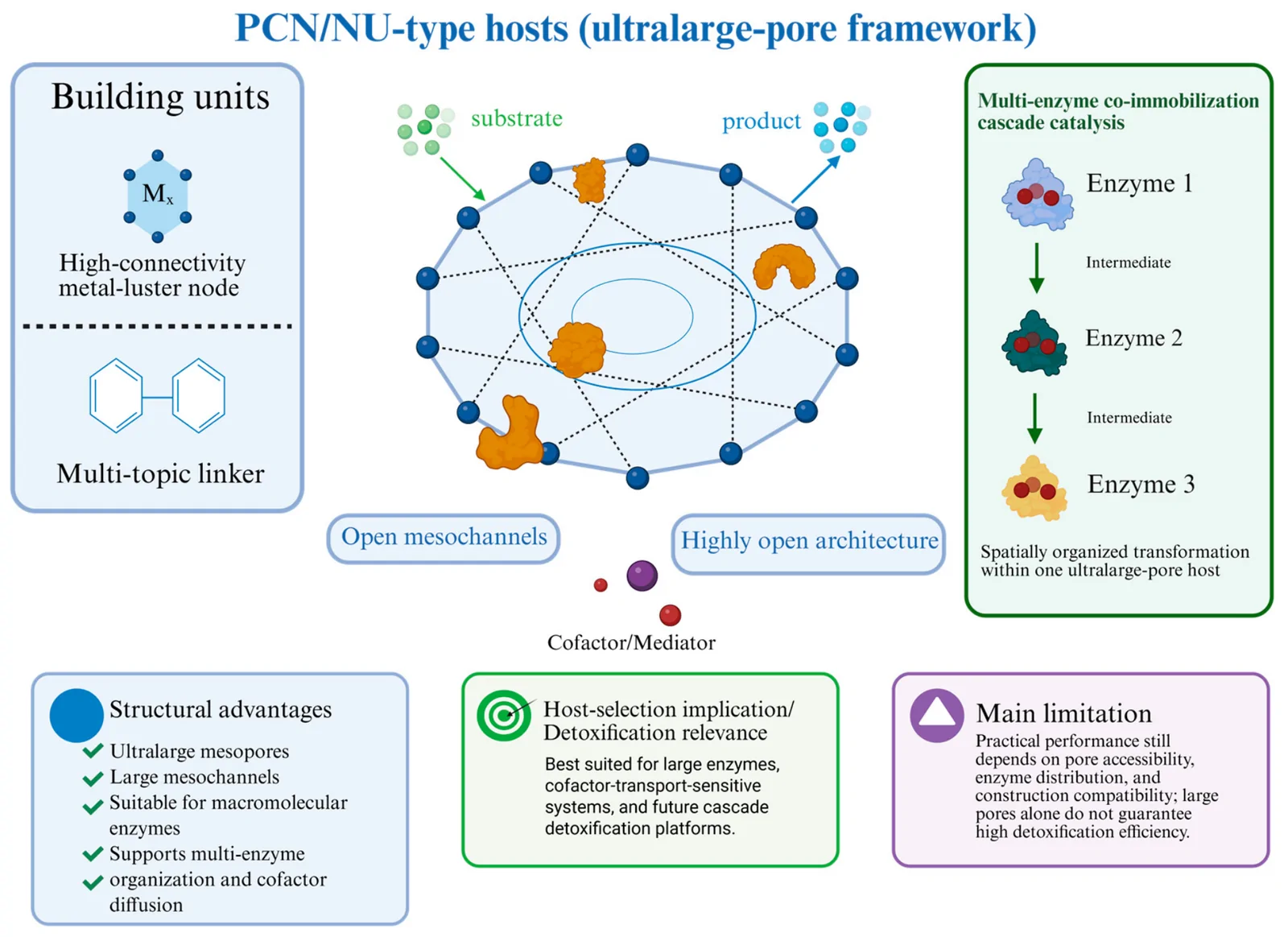
PCN/NU-type hosts for macromolecular enzyme accommodation and cascade catalysis.

Host selection is the next design step after choosing an immobilization mode. Different MOF families should not be compared only by surface area, pore volume, or prevalence of use, because their practical value depends on whether their framework features can solve the dominant bottleneck of a given toxin–enzyme–matrix system. Figure 5, Figure 6, Figure 7 and Figure 8 provide a host-selection map for four representative MOF families, highlighting their distinct strengths in enzyme encapsulation, structural robustness, interfacial regulation, and macromolecular or cascade catalysis. Therefore, this section discusses host selection from the perspective of detoxification requirements rather than material classification alone [20,21].

#### 5.2.1. ZIF-8-Type Hosts: Rapid Encapsulation for Protection-Dominated Systems

Figure 5 illustrates the typical structural and construction features of ZIF-8-type hosts, including Zn–imidazolate coordination, rapid nucleation, and mild aqueous synthesis. These features make ZIF-8 particularly suitable for one-pot enzyme encapsulation and biomimetic mineralization. For enzyme immobilization, the main value of ZIF-8 is not direct enzyme accommodation inside preformed micropores, but rapid formation of a protective MOF shell around biomacromolecules [48,74].

Accordingly, ZIF-8-type hosts are more appropriate for mycotoxin-degrading enzymes whose main limitation is activity loss during preparation, storage, or exposure to denaturing conditions. This host family is useful when rapid and mild encapsulation is required. However, the same structure shown in Figure 5 also defines its limitation. The intrinsic pore aperture of ZIF-8 is too small for most enzyme molecules, and excessive shell growth may restrict toxin diffusion or product release. Therefore, ZIF-8 should be selected for protection-dominated systems, but its application requires careful control of shell thickness, defect formation, substrate transport, and stability in aqueous, acidic, or matrix-rich environments [20,21,48,74].

ZIF-8 is constructed from ZnN_4_ tetrahedral nodes and 2-methylimidazolate linkers, featuring rapid nucleation under mild aqueous conditions. These properties make ZIF-8 suitable for surface adsorption and in situ biomimetic mineralization for constructing enzyme@ZIF-8 systems. Its main advantage lies in the formation of protective MOF shells, which can improve enzyme conformational stability during preparation, storage, and stress exposure. Therefore, ZIF-8 is particularly suitable for protection-dominated mycotoxin-degrading systems. However, its small intrinsic pore apertures generally limit direct accommodation of large enzymes; shell thickness, defect structure, and substrate/product diffusion must therefore be carefully controlled to avoid converting enzyme protection into mass-transfer resistance.

#### 5.2.2. UiO-Type Hosts: Robust Frameworks for Stability-Demanding Systems

Figure 6 emphasizes the structural basis of UiO-type hosts: Zr_6_ clusters, strong Zr–O coordination, and a chemically robust fcu framework. Compared with ZIF-8, UiO-type MOFs are less defined by rapid mineralization and more by framework stability and engineering flexibility. Functionalized linkers, defect sites, and hierarchical porosity can be used to regulate enzyme anchoring, pore accessibility, and the local microenvironment while maintaining relatively high structural robustness [75,81,82].

For mycotoxin detoxification, UiO-type hosts are most relevant when long-term operation, repeated use, or matrix resistance is required. They may be preferable when enzyme leakage, framework degradation, or medium-induced instability becomes the dominant bottleneck. However, the native microporosity of UiO-66 limits direct accommodation of large enzymes. Therefore, UiO-type hosts usually need to be combined with surface immobilization, defect engineering, mesoporous reconstruction, or in situ mineralization for large-enzyme systems. Their selection should be based on the need for robust operation and controllable interface engineering, rather than simple encapsulation capacity [75,81,82].

UiO-type MOFs are built from Zr_6_ cluster nodes and terephthalate-based linkers, and their strong Zr–O coordination provides high chemical and aqueous stability. Functionalized linkers, defect engineering, and hierarchical porosity can be used to regulate enzyme anchoring, interfacial interactions, and mass-transfer behavior, making UiO-type hosts suitable for immobilized detoxification systems requiring repeated use, matrix tolerance, and stable operation. Because the native microporosity of typical UiO-66 frameworks limits direct accommodation of large enzymes, surface immobilization, defect regulation, mesoporous reconstruction, or in situ mineralization is often required to balance enzyme stability with substrate accessibility during mycotoxin degradation.

#### 5.2.3. MIL-Type Hosts: Tunable Interfaces for Interaction-Regulated Systems

As illustrated schematically in Figure 7, MIL-101-type hosts are used here as representative large-cage MIL frameworks. These frameworks are characterized by relatively large cages, accessible pore windows, and modifiable pore-wall chemistry [73]. Such structural features make them suitable for systems in which enzyme loading, enzyme–carrier interactions, and substrate/product diffusion must be optimized together. In this context, MIL-101-type hosts are better regarded as interface-regulating platforms than simply as high-capacity enzyme carriers [83,84].

This selection logic is particularly important for mycotoxin-degrading enzymes because detoxification depends on whether toxin molecules can reach the catalytic site and whether transformation products can diffuse out of the catalytic microenvironment. Amino functionalization, linker modification, crosslinking, and composite construction can tune enzyme–carrier interactions and improve operational functions such as recovery and reuse [83,84]. Nevertheless, MIL-type hosts should not be treated as universally stable platforms. Their performance depends on metal composition, water stability, pore accessibility, and reaction medium. Therefore, they are most suitable when tunable interfacial chemistry and process-oriented functions of composite systems are required.

MIL-type MOFs, especially MIL-101-type frameworks, generally possess large cages, wide pore windows, and tunable pore-wall chemistry, which can facilitate enzyme loading, toxin access, and product release. Surface functionalization, crosslinking, and linker modification can further regulate enzyme–carrier interactions and pore microenvironments, thereby helping to balance enzyme immobilization, substrate accessibility, and product diffusion. Therefore, MIL-type hosts are suitable for mycotoxin-degrading systems in which mass transfer and interfacial interactions must be optimized simultaneously. However, their practical performance remains dependent on metal composition, framework stability, pore accessibility, and the reaction medium; large pores should not be directly equated with improved detoxification efficiency.

#### 5.2.4. PCN/NU-Type Hosts: Large-Pore Platforms for Macromolecular and Cascade Systems

Figure 8 highlights the defining advantage of PCN/NU-type hosts: ultralarge cages, open mesochannels, and hierarchical pore architectures. These structures provide sufficient space for loading macromolecular enzymes, multi-enzyme organization, and diffusion of substrates, products, mediators, or cofactors. Thus, PCN/NU-type hosts are particularly relevant when enzyme size, cofactor transport, multi-step transformation, or cascade catalysis becomes the main design challenge [44,45,76,85,86].

For future mycotoxin detoxification systems, this structural advantage is important because real food and feed contamination often involves multiple toxins, and some detoxification pathways may require sequential transformation of intermediates. Large-pore PCN/NU-type hosts may provide the spatial basis for organizing multiple catalytic units while maintaining molecular transport. However, large pores alone do not ensure high detoxification efficiency. Enzyme clustering, uneven distribution, channel blockage, or activity loss during immobilization may still compromise performance. Therefore, PCN/NU-type hosts should be regarded as promising platforms for macromolecular enzymes and cascade detoxification, but their practical value depends on controlled enzyme distribution, pore accessibility, compatibility of the construction process, and validation in real matrices [44,45,76,85,86].

PCN/NU-type MOFs are characterized by ultralarge mesopores, open mesochannels, and highly open framework architectures, with PCN-333(Al) and NU-1000 as representative hosts. These structural features provide sufficient spatial capacity for macromolecular enzyme loading, multi-enzyme co-immobilization, cofactor and mediator diffusion, and cascade catalysis, making PCN/NU-type hosts promising platforms for future multi-toxin or sequential detoxification systems. The figure illustrates the conceptual organization of multiple enzymes within one large-pore host for stepwise conversion of intermediates. Nevertheless, large pores alone do not guarantee efficient detoxification; practical performance still depends on enzyme distribution, pore accessibility, construction compatibility, cofactor/mediator transport, and stability in real food or feed matrices.

#### 5.2.5. From MOF Family Selection to Detoxification-Oriented Host Evaluation

The comparison of different MOF families indicates that host selection should move beyond material classification and toward detoxification-oriented functional evaluation. For mycotoxin-degrading enzyme@MOF systems, a suitable host is not defined simply by its ability to load enzymes or form stable composites. Instead, it should answer three key questions: whether the enzyme can retain an effective conformation within or on the host, whether toxins and products can efficiently access and leave the catalytic microenvironment, and whether the system can remain stable and safe in realistic food or feed matrices.

Therefore, the selection of ZIF-8-type, UiO-type, MIL-type, or PCN/NU-type hosts should be based on a stepwise evaluation framework. The first level is structural compatibility, including enzyme size, immobilization location, active-site exposure, and pore accessibility. The second level is catalytic compatibility, including toxin entry, product release, cofactor or mediator transport, and activity retention during reaction. The third level is application compatibility, including matrix interference, framework stability, enzyme leakage, metal-node release, reusability, and safety of transformation products. Only when these requirements are considered together can the structural advantages of MOF hosts be translated into practical detoxification performance.

From this perspective, Figure 5, Figure 6, Figure 7 and Figure 8 should not be regarded merely as structural illustrations of representative MOF families, but as a design map for host screening. ZIF-8-type, UiO-type, MIL-type, and PCN/NU-type hosts provide different starting points, but the final choice should depend on whether a given host can solve the limiting step of a specific toxin–enzyme–matrix system. Future host design should therefore shift from single-parameter optimization toward multi-factor evaluation that integrates enzyme protection, catalytic accessibility, mass-transfer efficiency, real-matrix stability, and safety validation.

### 5.3. Application Modes and Evidence Maturity: From Proof-of-Concept to Real-Matrix Validation

The application relevance of MOF-immobilized mycotoxin-degrading enzymes cannot be judged solely by maximum degradation efficiency or system complexity. Buffer and model reactions establish catalytic feasibility, whereas real food and feed matrices test whether enzyme activity, toxin accessibility, catalyst recovery, and framework stability can be maintained under practically relevant conditions. Scale-related experiments and continuous operation provide an additional dimension of validation but should not be regarded as equivalent to real-matrix performance.

Current MOF-based mycotoxin-control systems can be grouped into four principal modes: single-enzyme immobilization, multi-enzyme or composite biocatalytic systems, adsorption–degradation coupling, and detection–degradation integration. Their practical value depends on both functional design and validation depth. A relatively simple enzyme@MOF system tested in a real matrix may therefore be more application-relevant than a multifunctional platform demonstrated only in buffer.

#### 5.3.1. Single-Enzyme Immobilization: From Catalytic Feasibility to Real-Oil Validation

Single-enzyme immobilization provides the most direct route for evaluating whether MOF hosts improve enzyme stability, recovery, and operational durability. Model-system studies have demonstrated enhanced OTA degradation, reuse, and preliminary reaction-volume expansion using immobilized lipases or carboxypeptidases [87,88]. These results establish catalytic feasibility but do not demonstrate performance in actual food or feed matrices.

More recent studies have extended immobilized laccase systems into edible oils. Lac@ZIFs(IM) achieved 93% AFB1 degradation within 4 h in edible oil and was subsequently evaluated under continuous circulation [89]. This study combines real-matrix treatment with early process-oriented validation, although longer operation, transformation-product safety, oil-quality preservation, and material-residue control remain to be established.

Lac@Fe-BTC provides a broader safety-oriented example. The system removed both AFB1 and ZEN from peanut oil, retained activity during repeated use, and was further evaluated through product characterization and zebrafish toxicity testing [90]. This combination of real-matrix treatment, reusability, product analysis, and biological assessment provides stronger support for detoxification than studies reporting only parent-toxin reduction.

Direct numerical ranking among these systems would nevertheless be inappropriate because toxin concentration, enzyme dosage, reaction time, matrix composition, water availability, and analytical procedures differed among studies. More meaningful comparisons should be based on immobilized-enzyme, free-enzyme, and carrier-only controls evaluated under identical conditions.

#### 5.3.2. Multi-Enzyme and Composite Biocatalytic Systems: Broader Function but Limited Matrix Validation

Multi-enzyme immobilization may support sequential transformation, intermediate processing, or treatment of co-occurring mycotoxins. However, direct validation of MOF-organized natural-enzyme systems in real food and feed matrices remains limited.

Co-immobilized DADH and AKR13B3 on magnetic MOF-derived nanoflowers improved DON degradation relative to the corresponding free enzymes and enabled repeated magnetic recovery [80]. The study confirms the feasibility of dual-enzyme immobilization, but the current evidence is primarily catalytic and operational. Validation in contaminated grain or feed, together with product profiling, residual-toxicity assessment, and cofactor-transport analysis, is still required.

A more complex biocatalytic composite containing laccase, an Aspergillus niger crude-enzyme preparation, aminated mesoporous silica nanoparticles, and ZIF-8 achieved 96.7% AFB1 reduction under optimized conditions and 65.8% when applied at a mass ratio of 1% to moldy corn [91]. The moldy-corn experiment provides direct real-matrix evidence, but the two values should not be interpreted as a controlled quantitative measure of matrix inhibition because the experimental conditions were not identical.

The composite composition also limits mechanistic interpretation. Without purified-enzyme, individual-component, carrier-only, and inactivated-enzyme controls, the respective contributions of the different enzymes, toxin adsorption, and material-assisted transformation cannot be resolved. Future multi-enzyme systems should therefore be evaluated using enzyme-ratio controls, intermediate profiling, component-specific comparisons, and residual-toxicity testing.

#### 5.3.3. Adsorption–Degradation Coupling: Distinguishing Removal from Transformation

MOFs may enrich mycotoxins near immobilized enzymes and thereby facilitate catalytic treatment. However, a decrease in extractable parent toxin may result from adsorption, enzymatic transformation, or both. This distinction is essential because physical removal alone does not demonstrate detoxification.

The Fe-BTC@Lac@FB hybrid combined magnetic biochar, laccase, and an Fe-based MOF for AFB1 treatment in peanut oil [92]. The composite outperformed free laccase and retained useful performance during repeated use. Importantly, the thermally inactivated material still adsorbed 11.2 mg/g AFB1, confirming that physical adsorption contributed to toxin removal. Although oil-quality and cytotoxicity assessments did not indicate marked adverse effects, the relative contributions of adsorption and enzymatic transformation were not fully resolved.

MOF-235 provides a useful contrast. It rapidly removed multiple aflatoxins and ZEN from vegetable oils but functioned as an adsorbent rather than a natural-enzyme carrier [93]. This study therefore supports MOF-based toxin removal, but not enzyme-mediated degradation. Similarly, Pd@PCN-222 and related peroxidase-like materials should be classified as enzyme-mimetic catalytic platforms rather than natural enzyme@MOF systems [94].

For adsorption–degradation composites, carrier-only controls, inactivated-enzyme controls, desorption experiments, transformation-product identification, and residual-toxicity evaluation are required before toxin disappearance can be interpreted as verified detoxification.

#### 5.3.4. Detection–Degradation Integration: A Complementary Monitoring Strategy

Detection–degradation integration combines toxin recognition, analytical signal output, and catalytic treatment within a single platform. Direct evidence for this mode remains limited. A recent dual-emission Eu-MOF/g-C_3_N_4_ system was designed as a bifunctional platform for AFB1 detection and degradation, providing a representative example of material-based detection–degradation integration [95]. However, this system relies on luminescent sensing and photocatalytic or material-mediated treatment rather than an immobilized natural mycotoxin-degrading enzyme.

Functionally related MOF systems should not automatically be classified as detection–degradation platforms. For example, an enzyme-like MOF incorporated into a polymeric membrane enabled efficient AFB1 removal but did not integrate analytical detection with treatment [96]. It is therefore more appropriately regarded as an enzyme-mimetic membrane-removal system rather than direct evidence for detection–degradation integration.

These emerging platforms may support treatment monitoring and residual-toxin measurement, but they should not be treated as evidence that natural enzyme@MOF detoxification systems have reached the same level of functional integration. Their application value depends on whether the analytical signal remains reliable in complex matrices, whether transformation products interfere with detection, and whether changes in signal intensity correspond to chemical transformation and reduced toxicological risk rather than parent-toxin disappearance alone.

Detection–degradation integration should therefore be regarded as a complementary process-control direction. Its translation to natural enzyme@MOF systems will require the simultaneous validation of sensing accuracy, catalytic transformation, transformation-product safety, and performance in real food or feed matrices.

#### 5.3.5. Real-Matrix Gaps and Application-Level Evaluation

Current real-matrix evidence is concentrated mainly in edible-oil matrices, including peanut and other vegetable oils, together with a single moldy-corn system [89,90,91,92,93]. Natural enzyme@MOF and composite biocatalytic systems have maintained treatment performance in lipid-rich matrices; Lac@ZIFs(IM) was further evaluated under continuous circulation, whereas Lac@Fe-BTC and Fe-BTC@Lac@FB retained activity or treatment capacity during repeated use [89,90,92]. MOF-235 also achieved rapid removal of multiple mycotoxins from vegetable oils, but this system relies on physical adsorption rather than enzymatic transformation [93]. The moldy-corn study extends validation to a heterogeneous solid matrix, in which toxin distribution, catalyst–substrate contact, and interference from endogenous components are more difficult to control [91]. These studies therefore provide complementary rather than directly comparable evidence: oil-based experiments mainly establish compatibility with lipid-rich matrices, whereas the corn experiment provides preliminary evidence for treatment in a complex solid substrate.

The available data do not support direct numerical ranking across these matrices because the studies differed in toxin concentration, enzyme dosage, catalyst composition, reaction time, water availability, and analytical procedures. Results obtained under optimized conditions and in real matrices should likewise not be treated as matched measurements of matrix inhibition unless the experiments were conducted under otherwise identical conditions. The current literature therefore supports the feasibility of selected MOF-based systems in edible oils and moldy corn but does not establish which platform performs best across food and feed matrices.

Future studies should compare the free enzyme, immobilized enzyme, blank MOF, and inactivated-enzyme composite under matched toxin concentrations, enzyme dosages, reaction times, and matrix conditions. Parent-toxin reduction should be separated from adsorption and chemical transformation. Application-oriented assessment should also include transformation-product identification, residual-toxicity testing, matrix-quality evaluation, catalyst recovery, and, where relevant, measurement of metal-ion, linker, or particle residues.

Table 3 provides a comparative overview of representative natural enzyme@MOF, composite biocatalytic, enzyme-mimetic, and adsorption-only platforms, focusing on their construction/working modes, main advantages, reported study-specific performance, application-evidence levels, and key limitations.

Reported performance and comparative interpretation: The summarized values were obtained under non-equivalent experimental conditions and therefore represent study-specific performance rather than directly comparable intrinsic catalytic efficiencies. The listed advantages and limitations are review-level syntheses of the reported evidence, not standardized ratings assigned by the original authors.

Evidence level: A cumulative five-level framework is used to characterize application-evidence maturity: Level 1, proof-of-concept testing in buffer or model systems; Level 2, Level 1 plus operational evaluation, such as stability, recovery, or reuse; Level 3, validation in a defined real food or feed matrix; Level 4, Level 3 plus transformation-product identification and/or toxicological or other safety-related assessment; and Level 5, Level 4 plus process-relevant validation. Scale-up or continuous operation conducted only in model systems does not qualify for Level 5. The levels indicate the maturity of reported evidence, not regulatory approval, technical superiority, or commercial readiness.

Overall, the evidence summarized in Table 3 remains uneven across platform types. Natural enzyme@MOF and composite systems most directly support enzyme-mediated transformation, although adsorption may also contribute in hybrid systems. Enzyme-mimetic and adsorption-only platforms provide complementary evidence for material-mediated treatment or physical removal. Among the studies summarized here, none simultaneously addresses real-matrix performance, transformation-product safety, material-residue control, and process-relevant operation.

### 5.4. Current Bottlenecks and Rational Design Directions

Despite encouraging progress, MOF-immobilized mycotoxin-degrading enzymes remain largely at the proof-of-concept stage. The key challenge is no longer whether MOFs can immobilize enzymes, but whether the resulting systems can maintain catalytic function, structural integrity, and safety in realistic food and feed environments. Future development should focus on four bottlenecks: interfacial durability, matrix-compatible mass transfer, safety validation, and process-compatible fabrication [16,20,51].

#### 5.4.1. Interfacial Durability: Evaluating Long-Term Function Rather than Initial Loading

Many studies still emphasize enzyme loading, initial activity, or short-term cycling, but these indicators are insufficient for application-oriented detoxification. Weak adsorption may cause enzyme leakage, whereas overly strong coordination or covalent anchoring may restrict enzyme flexibility and reduce active-site accessibility. Therefore, an effective interface should not be judged by binding strength alone, but by whether it maintains enzyme conformation, catalytic accessibility, and activity retention during repeated or continuous operation [20,51,55].

Future studies should report enzyme leakage during washing and reaction, activity decay over repeated cycles, structural changes of the enzyme and MOF host, and performance under exposure to matrix components or continuous-flow conditions. For mycotoxin detoxification, these long-term indicators are more informative than a single initial degradation rate.

#### 5.4.2. Matrix-Compatible Mass Transfer: Verifying Toxin Access Under Realistic Conditions

The second bottleneck is mass transfer under realistic matrix conditions. MOF pores and shells can protect enzymes, but they may also restrict toxin diffusion and product release. This problem becomes more pronounced in oils, grains, feeds, fermentation media, or gastrointestinal-like environments, where proteins, lipids, polysaccharides, salts, organic acids, and other matrix components may block pores, alter interfacial interactions, or affect framework stability. This concern is consistent with previous findings that pore architecture, enzyme spatial organization, substrate and product diffusion, cofactor accessibility, and reaction medium can strongly affect enzyme@MOF performance [44,45,55].

Therefore, pore design should be evaluated by actual toxin accessibility rather than by surface area or pore volume alone. Hierarchical pores, defect engineering, and surface functionalization should be used to balance enzyme protection with substrate and product transport. Apparent kinetic parameters can provide useful indirect evidence for this balance: an increased apparent Km may indicate reduced substrate accessibility caused by pore, shell, or interfacial barriers, whereas a decreased apparent Km may reflect local toxin enrichment near the catalytic interface. Changes in kcat or catalytic efficiency should also be interpreted together with enzyme conformation, active-site exposure, and mass-transfer conditions rather than as isolated kinetic values. Ultimately, catalytic performance should be tested in real matrices instead of being inferred from buffer-based reactions.

#### 5.4.3. Product and Material Safety Validation: Proving Risk Reduction Rather than Toxin Disappearance

For food and feed detoxification, toxin disappearance is not equivalent to risk reduction. MOF-based systems must demonstrate not only a decrease in parent toxin levels, but also the safety of transformation products and the stability of the material itself. Potential risks include incomplete degradation and unknown products or products with higher toxicity [16,97]. MOF-related risks, including enzyme leakage, metal-node release, linker migration, particle residues, and residual synthesis reagents, should also be considered, especially when materials directly contact food or feed matrices [55].

Accordingly, safety validation should cover both transformation products and residual materials. Parent-toxin reduction should be accompanied by product identification, residual toxicity evaluation, and, when possible, in vivo validation. At the same time, MOF-related risks, including metal-ion leaching, linker migration, particle residues, and food and feed-contact compatibility, should be assessed together with catalytic performance. Before practical application claims can be made, product safety and material residues should be verified using complementary methods. These may include LC–MS/MS-based product analysis, mass-balance tracking, cell or animal toxicity assays, metal-ion leaching tests, and food/feed-contact compatibility assessment.

If only parent-toxin reduction is reported, the result should be described as removal or transformation evidence rather than verified detoxification. This distinction is particularly important for adsorption–degradation coupling and MOFzyme systems, where toxin disappearance may result from physical adsorption, catalytic transformation, or both. Therefore, future studies should avoid using degradation efficiency as the sole safety indicator and should instead evaluate whether the treated matrix shows reduced toxicological risk together with acceptable material-residue behavior.

#### 5.4.4. Process-Compatible Construction: Moving Beyond Laboratory Preparation

Another important limitation is process compatibility. Many enzyme@MOF composites are prepared under small-scale and carefully controlled laboratory conditions, whereas food and feed applications require reproducible batches, mild preparation, controllable particle size, easy recovery, and low residual material levels. Harsh solvents, long synthesis times, difficult separation, poor formulation stability, or uncontrolled particle residues may prevent translation even when laboratory degradation results are promising [49,50].

Future construction strategies should prioritize process-compatible preparation. Aqueous or solvent-reduced synthesis, scalable formulation, magnetic or membrane-assisted recovery, spray-drying, fixed-bed operation, and continuous-flow systems are particularly relevant. These routes should be judged not only by preparation convenience, but also by whether they preserve enzyme activity, maintain mycotoxin-control performance in real matrices, reduce material-residue risks, and allow repeated or continuous operation.

For feed applications, processing-related conditions should also be considered as practical constraints. Pelleting, storage, gastric acidity, intestinal release, and compatibility with feed additives or probiotics may all affect enzyme activity, MOF stability, and catalyst retention. A system that performs well in buffer may still fail during feed processing, storage, or gastrointestinal exposure. From this perspective, process-compatible construction should be evaluated together with matrix performance and residue control, rather than treated as a secondary formulation issue.

#### 5.4.5. Evidence-Guided Design and Validation of Enzyme@MOF Systems

Future development should integrate enzyme engineering, MOF host design, matrix adaptation, and safety assessment within a coordinated workflow. Enzyme engineering can improve intrinsic catalytic properties before immobilization [35,36], whereas MOF host and interface design can regulate enzyme retention, catalytic accessibility, and molecular transport [20,21,22]. These components should not be optimized independently; their suitability must be evaluated against the target toxin, catalytic pathway, intended matrix, and mode of catalyst deployment.

A rational development process should begin by identifying the dominant cause of performance loss under application-relevant conditions. Matched comparisons among the free enzyme, immobilized enzyme, blank MOF, and inactivated-enzyme composite can help distinguish enzyme inactivation from adsorption, diffusion resistance, enzyme leakage, or framework instability. Kinetic analysis, mass-balance assessment, transformation-product profiling, and material-residue measurements can then indicate whether improvement should focus on the enzyme, host structure, immobilization interface, or formulation.

System development should proceed through evidence-based decision points rather than through degradation efficiency alone. A candidate should advance from model-system evaluation to real-matrix and process-oriented testing only when it demonstrates a reproducible advantage over the relevant controls. Data-driven methods may assist in prioritizing enzyme–MOF combinations according to enzyme dimensions, pore accessibility, framework stability, and interfacial compatibility, but predicted combinations still require experimental verification. The priority is therefore to establish reproducible design and validation rules that connect molecular engineering with verified detoxification performance.

### 5.5. Industrial Translation, Regulatory Considerations, and Environmental Sustainability

The commercial viability of enzyme@MOF systems depends not only on catalytic performance but also on how the catalyst is deployed, recovered, regulated, and managed after use.

#### 5.5.1. Industrial Feasibility and Economic Viability

The intended mode of use largely determines process feasibility. Direct addition to food or feed may improve catalyst–toxin contact but increases concerns regarding particle residues and constituent migration. Recoverable formats, such as magnetic composites, membranes, and flow-through reactors, may reduce material carryover but require efficient separation, fouling control, and stable operation. Continuous-circulation treatment provides early evidence of process integration, although current studies remain below pilot scale [89].

Economic evaluation should consider treatment capacity, catalyst lifetime, recovery losses, product-quality effects, and cost per unit of treated matrix. General MOF production studies identify precursor costs, solvent use and recovery, synthesis yield, activation, and production scale as important cost drivers [98,99]. These findings cannot be transferred quantitatively to mycotoxin-degrading enzyme@MOF systems, which also involve enzyme production, immobilization, residue control, and catalyst recovery. Reuse is economically valuable only when retained catalytic performance offsets these additional costs.

#### 5.5.2. Regulatory Considerations

The applicable regulatory pathway will depend on the jurisdiction, intended use, exposure scenario, and whether the composite is retained in a recoverable food-contact or processing configuration or is intentionally introduced into food or feed. Regulatory reviews of agri-food nanotechnology and food-packaging nanomaterials indicate that assessment is application-specific and should consider material identity, intended exposure, migration of framework constituents or particles, and safety [100,101].

Where small or nanosized particles may be present, particle-size distribution, dissolution or degradation behavior, exposure, and toxicological properties require specific characterization [102]. Within the European Union, if an enzyme@MOF composite is intended for direct incorporation into animal feed and falls within the feed-additive regulatory framework, target-species safety and functional efficacy would need to be demonstrated according to the applicable assessment principles [103,104]. Regulatory suitability should therefore be evaluated for the complete enzyme@MOF composite under its intended conditions of use and cannot be inferred solely from the properties of the enzyme, metal node, or organic linker considered separately.

#### 5.5.3. Environmental Sustainability and End-of-Life Management

The environmental performance of enzyme immobilization should be assessed across the complete treatment process. Life-cycle studies of representative MOFs indicate that precursor production, synthesis route, solvent consumption, washing, activation, and energy demand can substantially influence environmental impacts [105,106]. These general findings provide useful evaluation principles but do not demonstrate that mycotoxin-degrading enzyme@MOF systems are inherently more sustainable than free enzymes or conventional carriers.

Catalyst reuse may reduce repeated material production, but this advantage depends on working lifetime, recovery efficiency, and regeneration requirements. Spent composites may contain retained toxins, transformation products, matrix components, metals, and organic linkers. End-of-life assessment should therefore consider regeneration, particle loss, contaminated wash water, toxin desorption, and the release of framework constituents. Where safe reuse is not feasible, disposal should prevent concentrated mycotoxins or material residues from entering water, soil, or feed chains.

## 6. Conclusions and Perspectives

MOF-immobilized mycotoxin-degrading enzymes provide a means of connecting toxin-specific transformation chemistry with controllable catalytic microenvironments. The contribution of this field extends beyond improving enzyme loading or stability: appropriately designed hosts can regulate enzyme retention, catalytic accessibility, molecular transport, and catalyst recovery. By organizing available knowledge around toxin structure, MOF–enzyme interfaces, host selection, construction strategy, and application evidence, this review provides an application-oriented framework for evaluating enzyme@MOF systems.

The current evidence, however, remains uneven. Most studies demonstrate individual advantages such as parent-toxin reduction, enhanced stability, or reusability, whereas relatively few combine real-matrix performance with transformation-product identification, residual-toxicity assessment, framework stability, and material-residue control. Differences in toxin concentration, enzyme dosage, carrier composition, reaction conditions, and analytical procedures also prevent reliable cross-study ranking. Parent-toxin disappearance should therefore not be regarded as verified detoxification unless adsorption, chemical transformation, and toxicological outcomes have been distinguished.

Translation will require a minimum evidence package rather than a single performance metric. This should include appropriate free-enzyme and carrier controls, confirmation of transformation products and reduced toxicity, preservation of matrix quality, acceptable enzyme and material residues, sufficient catalyst lifetime, and performance under process-relevant conditions. Economic viability, regulatory suitability, and end-of-life management must then be evaluated for the intended deployment format. Only systems that satisfy these combined catalytic, safety, material, and process requirements can progress from laboratory biocatalysts toward reliable mycotoxin-control technologies for food and feed applications.

## Figures and Tables

**Table 1:** Structure-guided detoxification targets and MOF-immobilization design requirements for representative mycotoxins.

Mycotoxin	Toxicity-Related Structural Motif	Enzymatic Transformation Target	Representative Enzyme Classes	MOF Design Implication	Safety Evaluation Focus	References
AFB1	Difuran moiety, C8=C9 double bond, and lactone-related structure	Oxidation, demethylation, furan-ring modification, lactone-related transformation	Laccases and other multicopper oxidases (MCOs); other oxidoreductases	Maintain catalytic-site accessibility and avoid excessive diffusion resistance. For oxidoreductase-type systems, the local oxidative microenvironment may need to be considered.	Product toxicity and residual hepatotoxic/carcinogenic risk	[16,17,18,19,20,21,22]
ZEN	Resorcylic acid lactone structure	Lactone-ring hydrolysis or oxidation/reduction	Lactonase, hydrolase, oxidoreductase	Preserve access to lactone-related transformation sites and maintain activity in lipid-rich or grain/feed matrices.	Reduction of estrogenic activity	[16,17,20,21,22,23,24]
DON	Trichothecene skeleton with epoxide group	De-epoxidation, oxidation/reduction, or epimerization-related transformation	De-epoxidase, dehydrogenase/reductase, oxidoreductase	Balance enzyme protection with diffusion of relatively polar small-molecule toxins and, when required, cofactors or mediators.	Residual ribotoxicity and intestinal toxicity	[16,17,18,20,21,22,25,26]
OTA	Amide bond linking isocoumarin and phenylalanine moieties	Amide hydrolysis	Carboxypeptidase, amidase, lipase	Provide a hydrolysis-compatible microenvironment while preserving substrate access and product release.	Safety of OTα and other hydrolysis products	[14,16,17,20,21,22]

**Table 2:** Construction strategy selection for MOF-immobilized mycotoxin-degrading enzymes.

Construction Strategy	Best-Fit Enzyme/System	Main Advantage	Key Limitation	Suggested Detoxification Scenario	Reference
Surface immobilization	Large enzymes or enzymes requiring exposed active sites	Simple construction, good substrate accessibility, convenient recovery	Possible enzyme desorption; sensitive to buffer and matrix conditions	Rapidly constructed recyclable systems when substrate access is more important than full encapsulation	[20,21,40,78]
Coordination anchoring	His-tagged or metal-binding recombinant enzymes	Selective anchoring and potential orientation control	Competition from buffers, organic acids, salts, or other ligands; dependence on accessible metal sites	Recombinant degrading enzymes where orientation control may improve active-site exposure	[65,66]
Covalent anchoring	Enzymes requiring reduced leakage or repeated use	Strong enzyme–carrier linkage and improved operational durability	Possible conformational restriction or active-site shielding	Long-term or continuous detoxification systems requiring low enzyme leakage	[70,72,73]
In situ encapsulation/biomimetic mineralization	Fragile enzymes sensitive to denaturing conditions	Confinement protection and improved stress tolerance	Diffusion barriers may arise from shell thickness, narrow pore windows, or poor pore connectivity	Systems where enzyme protection is the primary bottleneck, provided toxin/product diffusion is maintained	[48,74,75]
Pore infiltration/hierarchical confinement	Enzymes requiring both protection and mass transfer	Balances enzyme accommodation with substrate/product diffusion	Requires matched pore size, pore connectivity, and host stability	Complex-matrix detoxification where toxin access and enzyme protection must be balanced	[44,45]
Co-immobilization/cascade construction	Multi-enzyme or multi-step transformation systems	Spatial cooperation and potential intermediate channeling	Enzyme ratio, diffusion path, intermediate accumulation, and cofactor/mediator transport are difficult to control	Multi-toxin or sequential detoxification systems; still a developing strategy requiring real-matrix and product-safety validation	[79,80]

**Table 3:** Comparative assessment of representative MOF-based platforms for mycotoxin control: main advantages, reported performance, application-evidence level, and key limitations.

Toxin	System and MOF Platform	Construction/Working Mode	Main Advantage	Reported Performance and Application Evidence	Evidence Level	Key Limitation	Reference
OTA	Amano lipase A immobilized on Zn-MOF (ANL@Zn-MOF)	One-step in situ immobilization	One-step construction with reuse and preliminary reaction-volume expansion	Under the specified model-system conditions, a reported OTA degradation of 100% was achieved within 8 h using ANL@Zn-MOF containing 20 ng/mL ANL. In a 500 mL reaction system, 69.3% OTA degradation was achieved within 12 h, and 78% of the initial activity was retained after five reuse cycles.	Level 2	Model-system and preliminary scale-related evidence; real-matrix validation, product identification, and toxicity assessment were not reported.	[87]
AFB1	Laccase immobilized in an imidazole-ligand ZIF (Lac@ZIFs(IM))	Ligand-screened in situ immobilization and continuous-flow operation	Real-oil validation combined with continuous-flow evaluation	Lac@ZIFs(IM) achieved 93% AFB1 degradation within 4 h in edible oil. During continuous operation, its activity after 20 h was reported to be 6.6 times that of Lac@ZIF-8.	Level 3	Transformation-product identification, toxicity attenuation, oil-quality assessment, and material-residue measurements were not reported.	[89]
AFB1 and ZEN	Laccase encapsulated in Fe-BTC (Lac@Fe-BTC)	In situ encapsulation	Treatment of two mycotoxins in peanut oil with product and toxicological assessment	AFB1 and ZEN removal in peanut oil reached 71.64% and 60.98%, respectively. After four reuse cycles, the corresponding removal rates were 52.47% and 46.82%. Transformation products were structurally characterized, and toxicity attenuation was reported in zebrafish assays.	Level 4	Evidence was obtained in peanut oil under study-specific conditions; validation in additional matrices, longer-term operation, and material-residue measurements were not reported.	[90]
DON	Co-immobilized DADH and AKR13B3 on magnetic MOF-derived nanoflowers	Ultrasound-assisted co-immobilization	Dual-enzyme organization combined with magnetic recovery and reuse	The optimized co-immobilized system showed a reported DON degradation level 1.1 times that of the free-enzyme system and retained 52% of its initial activity after ten reuse cycles.	Level 2	The reported evidence was obtained mainly in a model system; real grain or feed matrix performance, transformation-product profiles, and residual toxicity were not reported.	[80]
AFB1	Laccase/*Aspergillus niger* crude-enzyme biological detoxification polymer containing ZIF-8	In situ co-immobilization with aminated mesoporous silica nanoparticles	Integration of enzyme immobilization, toxin adsorption, reuse, and moldy-corn application	AFB1 reduction reached 96.7% at 35 °C after 24 h, and 65.8% reduction was achieved in moldy corn using 1% BDP. After four reuse cycles, enzyme activity and AFB1 reduction remained at 80.7% and 80.0%, respectively. Three transformation products were detected.	Level 4	The contributions of laccase, crude-enzyme components, ZIF-8 adsorption, and other carrier effects were not quantitatively separated. Reduced toxicity of the reported transformation products was not separately verified.	[91]
AFB1	Fe-BTC@Lac@FB natural enzyme@MOF–magnetic biochar hybrid	Laccase immobilization on magnetic biochar followed by Fe-BTC coating	Combination of enzymatic treatment, adsorption, magnetic recovery, and reuse in peanut oil	The reported AFB1 degradation was 11 times that obtained with free laccase. Thermally inactivated Fe-BTC@Lac@FB adsorbed 11.2 mg/g AFB1 from peanut oil, and more than 85% of the initial AFB1 removal efficacy was retained after five reuse cycles. Oil-quality parameters and cytotoxicity were evaluated, with low cytotoxicity reported under the assay conditions.	Level 4	Adsorption and enzymatic transformation were not quantitatively separated, and the transformation products were not identified.	[92]
AFB1	Pd@PCN-222 enzyme-mimetic system	Adsorption combined with H_2_O_2_-assisted peroxidase-like catalysis	Coupling of adsorption and material-mediated catalysis with reuse evaluation	Pd@PCN-222 removed 96.52% of AFB1 within 2 h under the reported experimental conditions, and material reusability was evaluated.	Level 2	This is an H_2_O_2_-dependent enzyme-mimetic system rather than an immobilized natural enzyme. Transformation products, reaction by-products, residual toxicity, and real-matrix performance were not reported.	[94]
AFB1, AFB2, AFG1, AFG2, and ZEN	MOF-235 adsorption system	Adsorption treatment in vegetable oils	Simultaneous removal of multiple mycotoxins from a real oil matrix	MOF-235 removed more than 96.1% of the investigated aflatoxins and 83.3% of ZEN from vegetable oils within 30 min. Low cytotoxicity was reported for the treated-oil samples under the assay conditions, and material reusability was evaluated.	Level 4	Removal was achieved through adsorption rather than enzymatic or chemical transformation. This system therefore provides real-matrix removal evidence but not evidence of enzyme-mediated detoxification	[93]

## Data Availability

The original contributions presented in this study are included in the article. Further inquiries can be directed to the corresponding author(s).
